# Genome-wide SNP data reveal phylogenetic relationships and further new species of lace bugs (Hemiptera, Heteroptera, Tingidae) endemic to the Japanese oceanic islands (the Ogasawara Islands), providing insight into their speciation

**DOI:** 10.3897/zookeys.1294.205145

**Published:** 2026-10-08

**Authors:** Jun Souma, Naoyuki Nakahama

**Affiliations:** 1 Shirakami Research Center for Environmental Sciences, Faculty of Agriculture and Life Science, Hirosaki University, Aomori, Japan Shirakami Research Center for Environmental Sciences, Faculty of Agriculture and Life Science, Hirosaki University Aomori Japan; 2 Department of Environmental Sciences and Technology, Graduate School of Agriculture, Osaka Metropolitan University, Osaka, Japan Department of Environmental Sciences and Technology, Graduate School of Agriculture, Osaka Metropolitan University Osaka Japan

**Keywords:** Adaptive radiation, East Asia, geographic isolation, host shift, MIG-seq, Oceanian Region, Ogasawara Islands, phytophagous insect

## Abstract

The Ogasawara Islands of Japan are oceanic islands that are home to two endemic genera of lace bugs (Hemiptera, Heteroptera, Tingidae), namely, *Acanthomoplax* Souma & Kamitani, 2021 (Tinginae, Tingini) and *Omoplax* Horváth, 1912 (Tinginae, Tingini). In the present study, phylogenetic analysis, using genome-wide single-nucleotide polymorphism (SNP) data from multiplexed inter-simple sequence repeat (ISSR) genotyping by sequencing (MIG-seq), is conducted for the genus *Omoplax*, which is hypothesized to have undergone adaptive radiation in the Ogasawara Islands. Additionally, population genetic analysis using these data is conducted for *O. desecta*, for which two host races are known. Combining the results of these analyses with the morphological differences, *O. sandoru* Souma, **sp. nov**., previously identified as the population in Hahajima Group of *O. desecta*, is described as a new species. In conclusion, the following nine lace bug species are recognized in the Ogasawara Islands: *A. tomokunii* Souma & Kamitani, 2021, *O. desecta* (Horváth, 1912), *O. hisasuei* Souma, 2025, *O. inugusu* Souma, 2025, *O. karubei* Souma, 2022, *O. kobugashi* Souma, 2025, *O. majorcarinae* Guilbert, 2001, *O. mukojimensis* Souma, 2022, and *O. sandoru* Souma, **sp. nov**. An updated key for identifying all nine species of lace bugs occurring in the Ogasawara Islands is provided. Finally, insights into phylogenetic relationships and the mechanism of speciation within the genus *Omoplax* are discussed.

## Introduction

On oceanic islands, many endemic animal and plant species are known because of their isolation from the continent (Karube and Takakuwa 2004; Cowie and Holland 2008; Kier et al. 2009). Unfortunately, many of them are currently threatened with extinction due to human activity and the impact of invasive organisms, and some have already become extinct (Krushelnycky et al. 2005; Cowie and Holland 2008; Fernández-Palacios et al. 2021; Yoshino 2025). The Ogasawara Islands (also known as the Bonin Islands), the only part of Japan classified in the Oceanian Region, comprise more than 100 oceanic islands and rocks located approximately 900–1,000 km south of the mainland of Tokyo Metropolis, Honshu, Japan (cf. Shimizu 2003; Karube and Takakuwa 2004). As with other islands, the Ogasawara Islands have an exceptionally high proportion of endemic organisms, including over 90% of land snails, 30% of insects, and 40% of plants (Toyoda 1981; Kato 1992; Tomiyama 1992a; Shimizu 2003; Karube and Takakuwa 2004). Additionally, evolutionary phenomena unique to oceanic islands, i.e., pronounced adaptive radiation among land snails and plants and the evolution of dioecy and woodiness in plants, also characterize these islands (Shimizu and Tabata 1991; Tomiyama 1992b; Shimizu 2003; Karube and Takakuwa 2004; Chiba 2010). Based on findings of the evolutionary biological studies, the Ogasawara Islands were inscribed on the UNESCO World Natural Heritage site under the criteria (ix) “to be outstanding examples representing significant on-going ecological and biological processes in the evolution and development of terrestrial, fresh water, coastal and marine ecosystems and communities of plants and animals” (UNESCO World Heritage Centre 1992–2026; Ministry of the Environment 2026).

In contrast, approximately 440 endemic insect species have been reported in the Ogasawara Islands (Hisasue 2026). However, these are not recognized as valuable to the Ogasawara Islands as a World Natural Heritage site because of a lack of evolutionary biological studies. Moreover, many endemic insects are at risk of extinction owing to predation by invasive animals, such as the green anole *Anolis carolinensis* Voight, 1832 (Squamata, Dactyloidae) and the nemertean worm *Geonemertes* sp. (Monostilifera, Prosorhochmidae) (Karube and Takakuwa 2004; Makihara et al. 2004; Karube 2005; Shinobe et al. 2017; Yoshino 2025). To date, evolutionary biological studies have determined sister species distributed in other regions of some insect species endemic to the Ogasawara Islands (Sota et al. 2011; Huang et al. 2019; Nagata et al. 2021; Chen et al. 2026). However, the endemic insect taxa that appear to have undergone adaptive radiation in the Ogasawara Islands have been poorly studied in evolutionary biology. As previous studies on land snails and plants have demonstrated, understanding the mechanisms of speciation among these endemic insect taxa is expected to enhance the value of the Ogasawara Islands as a World Natural Heritage site.

Phytophagous insects and their mechanisms of speciation have been extensively studied, with geographic isolation and host shift known to increase speciation rates (e.g., Matsubayashi et al. 2010; Zelvelder et al. 2026). Lace bugs (Hemiptera, Heteroptera, Tingidae) are phytophagous and often highly host specific (Schuh and Weirauch 2020) and include numerous species endemic to oceanic islands (cf. Drake and Poor 1943; Froeschner 1976; Guilbert 2001). Currently, eight species belonging to two endemic genera, *Acanthomoplax* Souma & Kamitani, 2021 (Tinginae, Tingini) and *Omoplax* Horváth, 1912 (Tinginae, Tingini), are known from the Ogasawara Islands (Souma 2025): *A. tomokunii* Souma & Kamitani, 2021, *O. desecta* (Horváth, 1912), *O. hisasuei* Souma, 2025, *O. inugusu* Souma, 2025, *O. karubei* Souma, 2022, *O. kobugashi* Souma, 2025, *O. majorcarinae* Guilbert, 2001, and *O. mukojimensis* Souma, 2022. Host plants are known for seven species, with the host of *O. mukojimensis* not known (Souma 2025). All species except *A. tomokunii* and *O. desecta* are endemic to one of the three island groups, namely, Mukojima, Chichijima, and Hahajima, of the Ogasawara Islands (Souma 2025). Furthermore, the following three pairs of *Omoplax* species that feed on the same or congeneric host plants are allopatrically distributed in island groups or islands (Souma 2025): *O. desecta* and *O. karubei* feeding on Rhaphiolepis indica (L.) Lindl. var. tashiroi Hayata (Rosales, Rosaceae); *O. hisasuei* and *O. majorcarinae* feeding on *Neolitsea sericea (Blume) Koidz. var. aurata* (Hayata) Hatus. (Laurales, Lauraceae); and *O. inugusu* and *O. kobugashi* feeding on *Machilus* species (Lauraceae). Thus, the genus *Omoplax* can be hypothesized to have undergone adaptive radiation in the Ogasawara Islands. Meanwhile, *O. desecta* feeds on R. indica var. tashiroi in Chichijima and Hahajima groups, and on *Calophyllum inophyllum* L. (Malpighiales, Calophyllaceae) only in Chichijima Group (cf. Souma 2025). The population in Chichijima Group of *O. desecta* sometimes exhibits color variation not found in the population in Hahajima Group. Therefore, the two host races and/or the populations between the two island groups of *O. desecta* may be in the process of speciation. We therefore considered lace bugs of the Ogasawara Islands excellent candidates for an in-depth evolutionary investigation.

Evolutionary biological studies of Tingidae are required to recognize endemic insects as valuable to the Ogasawara Islands as a World Natural Heritage site. In the present study, we conducted population genetic and phylogenetic analyses using genome-wide single-nucleotide polymorphism (SNP) data from multiplexed inter-simple sequence repeat (ISSR) genotyping by sequencing (MIG-seq) (Suyama and Matsuki 2015; Suyama et al. 2022) to understand the mechanism of speciation within the genus *Omoplax*. In addition, we reassessed the systematics and distribution of *Omoplax* species feeding on *C. inophyllum* and R. indica var. tashiroi.

## Material and methods

### MIG-seq

A total of 52 samples from eight Japanese tingid species were used in the present study (Suppl. material 1). All samples were identified based on their morphological characteristics before DNA extraction. The 41 samples comprising only *Omoplax desecta*, as identified based on previous studies (e.g., Souma and Kamitani 2021; Souma 2022a, 2025), which included *O. sandoru* sp. nov., were used in population genetic analyses to reveal variations within and between species. The 28 samples comprising eight lace bug species were used in the phylogenetic analysis to estimate phylogenetic relationships within the genus *Omoplax*. Of these 28 samples, 27 samples were composed of seven of the eight recognized *Omoplax* species and were used as ingroups. Because no specimens other than the holotype, from which DNA cannot be extracted (cf. Souma 2022a, 2025), *O. mukojimensis* was excluded from the phylogenetic analysis. The remaining sample, Stephanitis (Norba) exigua Horváth, 1912, was used as the outgroup. S. (N.) exigua suck sap from the abaxial side of the leaves of evergreen broad-leaved trees, similar to *Omoplax* species, and is endemic to the Ryukyu (Okinawa Group) and Daito Islands of Japan, which are located at a similar latitude to the Ogasawara Islands (Souma 2022b, 2025). The genus *Omoplax* was originally described as a subgenus of *Stephanitis* Stål, 1873, because of superficial similarities in structure (Horváth 1912), but was later upgraded to genus level based on morphological differences in the paranotum (Takeya 1962). All sequences used in the present analyses have been deposited in the Sequence Read Archive of the DNA Data Bank of Japan (DDBJ) under the accession numbers PRJNA1477673 (BioProject) and SAMN60765438–SAMN60765489 (BioSample).

DNA was extracted from the whole body, from the hemelytra and legs, from the head and thorax, or from the abdomen, using either the DNeasy Blood & Tissue Kit (Qiagen, Hilden, Germany) or the Chelex method (Miura et al. 2017). The PCR-amplified DNA of 52 samples, consisting of 63,948–782,974 short sequences, was then subjected to multiplexed inter-simple sequence repeat (ISSR) genotyping by sequencing (MIG-seq) (Suyama and Matsuki 2015; Suyama et al. 2022) to perform population genetic and phylogenetic analyses. The first PCR, the second PCR, and the NGS run were conducted in accordance with the protocols provided in MIG-seq ver. 4.1 (Suyama et al. 2022). Library preparation and sequencing were performed on a MiSeq (Illumina, San Diego, CA, USA) at GENODAS (Miyagi, Japan).

Low-quality reads and primer sequences were eliminated from the sequenced raw data using Trimmomatic ver. 0.39 (Bolger et al. 2014). The quality-filtered sequence data were demultiplexed and filtered for the SNP datasets of the 41 and 28 samples using Stacks ver. 2.62 (Catchen et al. 2013; Rochette et al. 2019) with “denovo_map.pl” by the following option settings recommended by Suyama et al. (2022): minimum depth of coverage required to create a stack (m) = 3; maximum distance allowed between stacks (M) = 2; and maximum distance allowed to align secondary reads to primary stacks (N) = 2.

For a population genetic analysis of *O. desecta*, as identified based on previous studies (e.g., Souma and Kamitani 2021; Souma 2022a, 2025), the “populations” in Stacks ver. 2.62 was used for the SNP dataset of 41 samples with the following option settings: minimum number of populations in a locus (p) = 1; a minimum minor allele count (min_mac) = 3; and maximum observed heterozygosity (max_obs_het) = 0.6; and data analysis restricted to a single SNP per locus (write_single_snp). The SNP dataset of the 41 samples was filtered using TASSEL ver. 5.2.96 (Bradbury et al. 2007) to a proportion of missing gametes < 0.30 by the following option setting: site minimum count of alleles not unknown = 25. A total of 673 single-nucleotide polymorphisms (SNPs) were detected by filtering, with the proportion of missing gametes = 0.24419. A distance-based phylogenetic network was inferred using the SplitsTree App ver. 6.0.0 (Huson and Bryant 2024). The P Distance method (Hamming 1950) was used with default options to obtain a 41 × 41 editDistance matrix. The Neighbor-Net method (Bryant and Moulton 2004; Bryant and Huson 2023) was used with default options to obtain 108 cyclic splits. The Show Splits method was used with default options to obtain a Split Network visualization. As an alternative approach to represent the genetic relationships among the 41 samples, Principal Component Analysis (PCA) using PC1 versus PC2 was performed by TASSEL ver. 5.2.96 on the 673 SNPs. The PCA result was visualized using Microsoft Excel (Microsoft Corp., Washington, USA).

For the phylogenetic analysis of eight tingid species, the “populations” in Stacks ver. 2.62 was also used for the SNP dataset of the 28 samples with the following option settings: minimum proportion of individuals required to process a locus across all data (r) = 0.2; minimum number of populations in a locus (p) = 1; and a minimum minor allele count s (min_mac) = 3. A phylogenetic tree based on the detected dataset of 4,452 SNPs was constructed using the Maximum Likelihood (ML) method in IQ-TREE3 ver. 3.1.2 (Wong et al. 2026), using the GTR+ASC model. Shimodaira-Hasegawa-like approximate likelihood ratio (SH-aLRT) support values and Ultrafast bootstrap (UFBoot) support values were assessed using 1,000 replicates. The tree was visualized using FigTree ver. 1.4.4 (Rambaut 2014).

All illustrations of the analyses were processed using Adobe Illustrator 2026 ver. 30.4 (Adobe Inc., San Jose, CA, USA) and Adobe Photoshop 2026 ver. 27.7.

### Systematics

Morphological characteristics of the specimens were observed, drawn, and measured under a stereoscopic microscope (SZX16; Olympus, Tokyo, Japan) equipped with an ocular grid. To examine the male genitalia, the pygophore was removed from the body after softening the specimens in hot water. The removed pygophore was immersed in hot 15% potassium hydroxide (KOH) solution for 5 min. The paramere was immersed in 99% ethanol and removed from the pygophore for further observation. Male genitalia were observed after the angles were fixed with gel (Museum Gel Clear; Ready America, California, USA) and placed on a microscope slide. Male genitalia were preserved in small polyethylene vials containing a 50% aqueous glycerin solution. A polyethylene vial was mounted on the pin containing the specimens. The specimens were photographed using a digital camera (EOS 90D; Canon, Tokyo, Japan) with a macro lens (MP-E65 mm F2.8 1–5×; Canon, Tokyo, Japan) and a digital microscope (Dino-Lite Premier M; Opto Science, Tokyo, Japan). Photographs of host plants were captured using a smartphone (iPhone 14; Apple, California, USA). The image stacks of the specimens were processed using a Zerene Stacker (Zerene Systems, Washington, USA). All illustrations and photographs were processed using Adobe Illustrator 2026 ver. 30.4 and Adobe Photoshop 2026 ver. 27.7. Morphological terms were assigned according to previous monographs (Takeya 1962; Drake and Davis 1960; Drake and Ruhoff 1965; Schuh and Weirauch 2020).

The specimens examined in the present study have been deposited at the National Museum of Nature and Science, Ibaraki, Japan (**NSMT**), and the Shirakami Research Center for Environmental Sciences, Faculty of Agriculture and Life Science, Hirosaki University, Aomori, Japan (**SIHU**). In the present study, all lace bug specimens recorded as *Omoplax desecta* in a recent study (Souma 2025) were reexamined. However, the Results section only mentions specimens that were previously misidentified.

Distribution maps for each species were created using data from the Geospatial Information Authority of Japan (GSI) (https://maps.gsi.go.jp) and were edited using Adobe Photoshop. Scientific names of the host plants were assigned according to Yonekura and Kajita (2003–2026).

## Results

### MIG-seq

**Figure 1.**
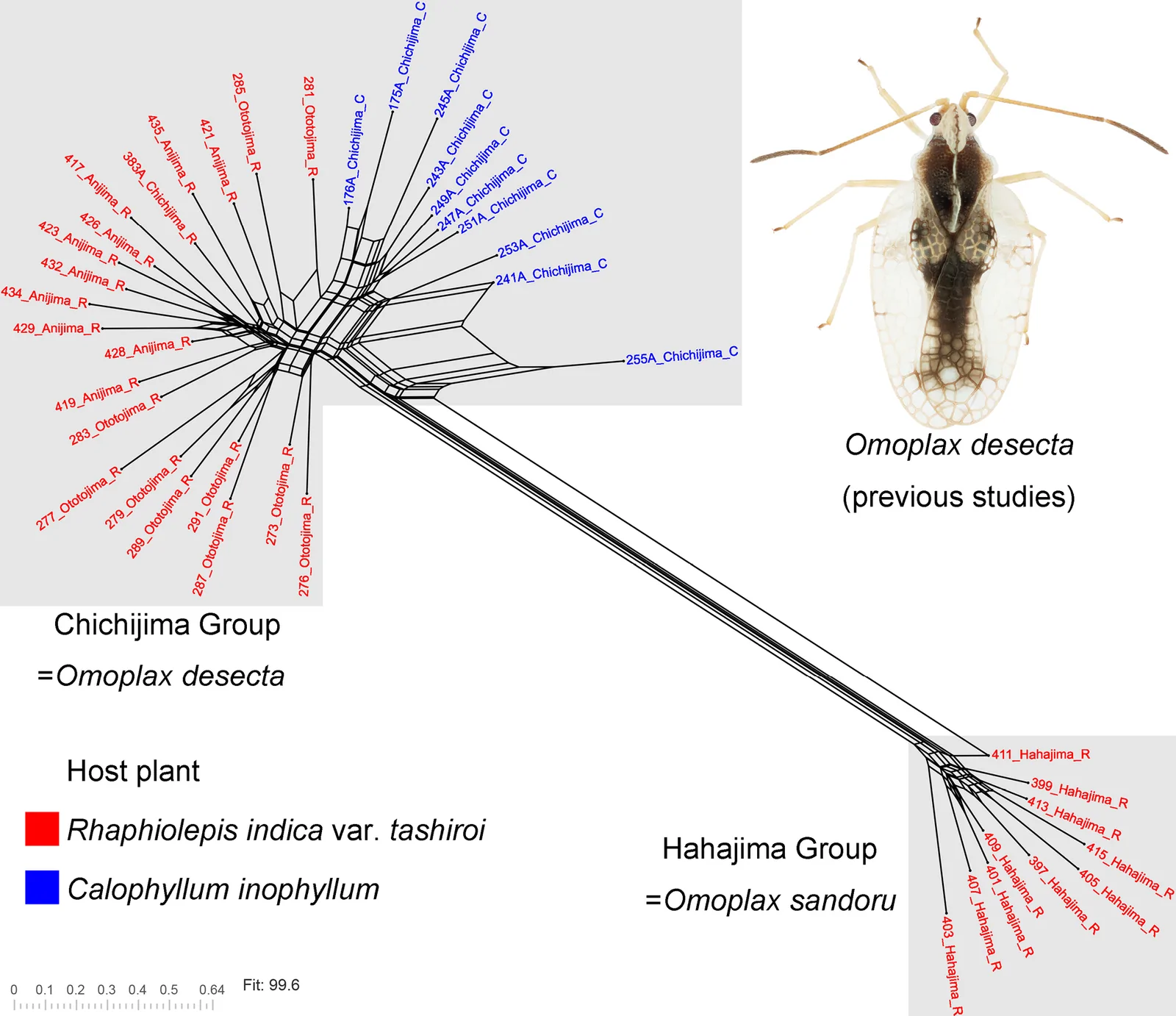
Neighbor-Net network obtained from SplitsTree based on 673 single-nucleotide polymorphisms (SNPs) of *Omoplax desecta*, as identified based on previous studies. The scale bar represents the uncorrected *p*-distance (the proportion of nucleotide differences per site) between samples. The Fit value (99.6%) indicates the percentage of variance in the distance matrix explained by the network. Higher values reflect a closer correspondence between the observed pairwise distances and the Neighbor-net network. Each sample ID includes the island name of the collection site.

The Neighbor-Net network of 673 single-nucleotide polymorphisms (SNPs) explained 99.6% of the variance in the pairwise distance matrix (*Fit* = 99.6), indicating a close correspondence between observed genetic distances and the inferred splits (Fig. 1). *Omoplax desecta*, as identified based on previous studies (e.g., Souma and Kamitani 2021; Souma 2022a, 2025), strongly clustered into the two Chichijima and Hahajima groups, and weakly clustered according to host plant. However, *O. desecta* of Chichijima Group did not cluster into populations on the islands of Anijima, Chichijima, or Ototojima.

**Figure 2.**
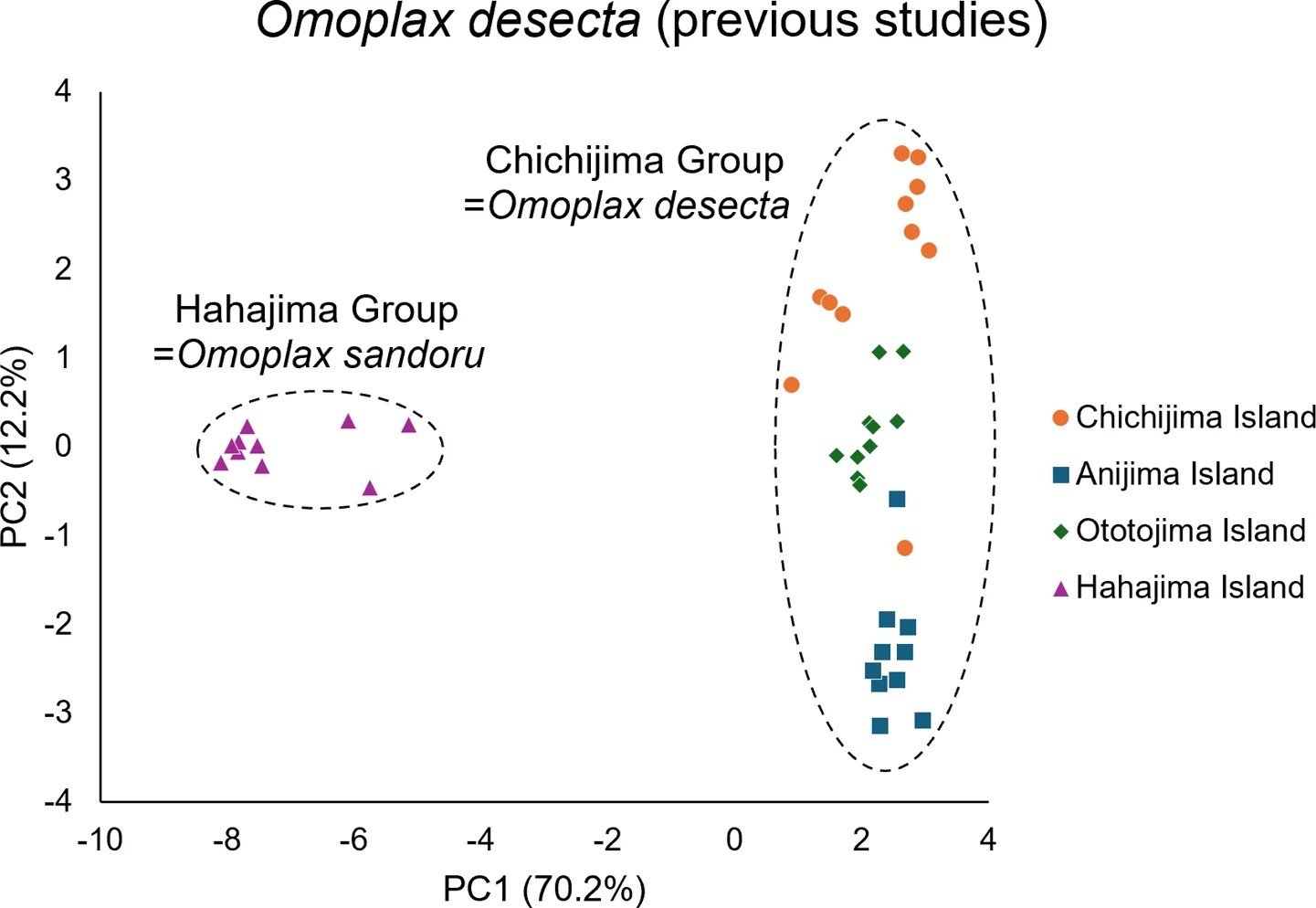
Principal Component Analysis (PCA) performed in TASSEL using PC1 versus PC2 based on 673 SNPs of *Omoplax desecta*, as identified based on previous studies.

The first two Principal Components (PCs) were generated via Principal Component Analysis (PCA) based on 673 SNPs and explained 82.4% of the total variation (Fig. 2). These PCs detected two genetic subgroups, each comprising populations in Chichijima and Hahajima groups. In concordance with the Neighbor-Net network results, no genetic subgroups were detected among the three populations on the islands of Anijima, Chichijima, or Ototojima.

**Figure 3.**
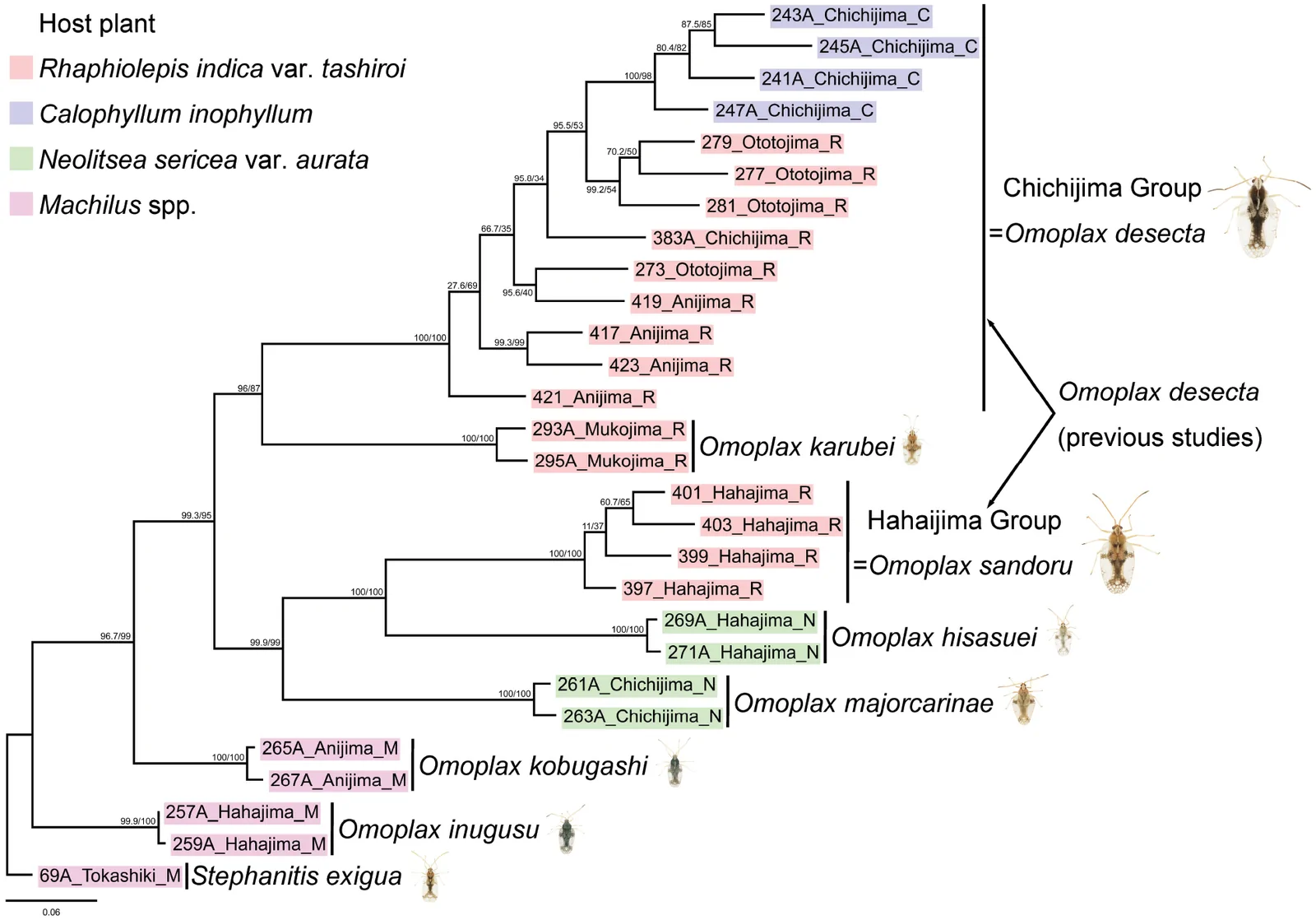
Phylogenetic tree obtained from an IQ-TREE Maximum Likelihood (ML) analysis based on 4,452 SNPs, showing relationships within the genus *Omoplax* endemic to the Ogasawara Islands of Japan. The numbers associated with the nodes represent Shimodaira-Hasegawa-like approximate likelihood ratio (SH-aLRT) support values and Ultrafast bootstrap (UFBoot) support values. The scale bar represents the expected number of substitutions per site. Each sample ID includes the island name of the collection site.

**Figure 4.**
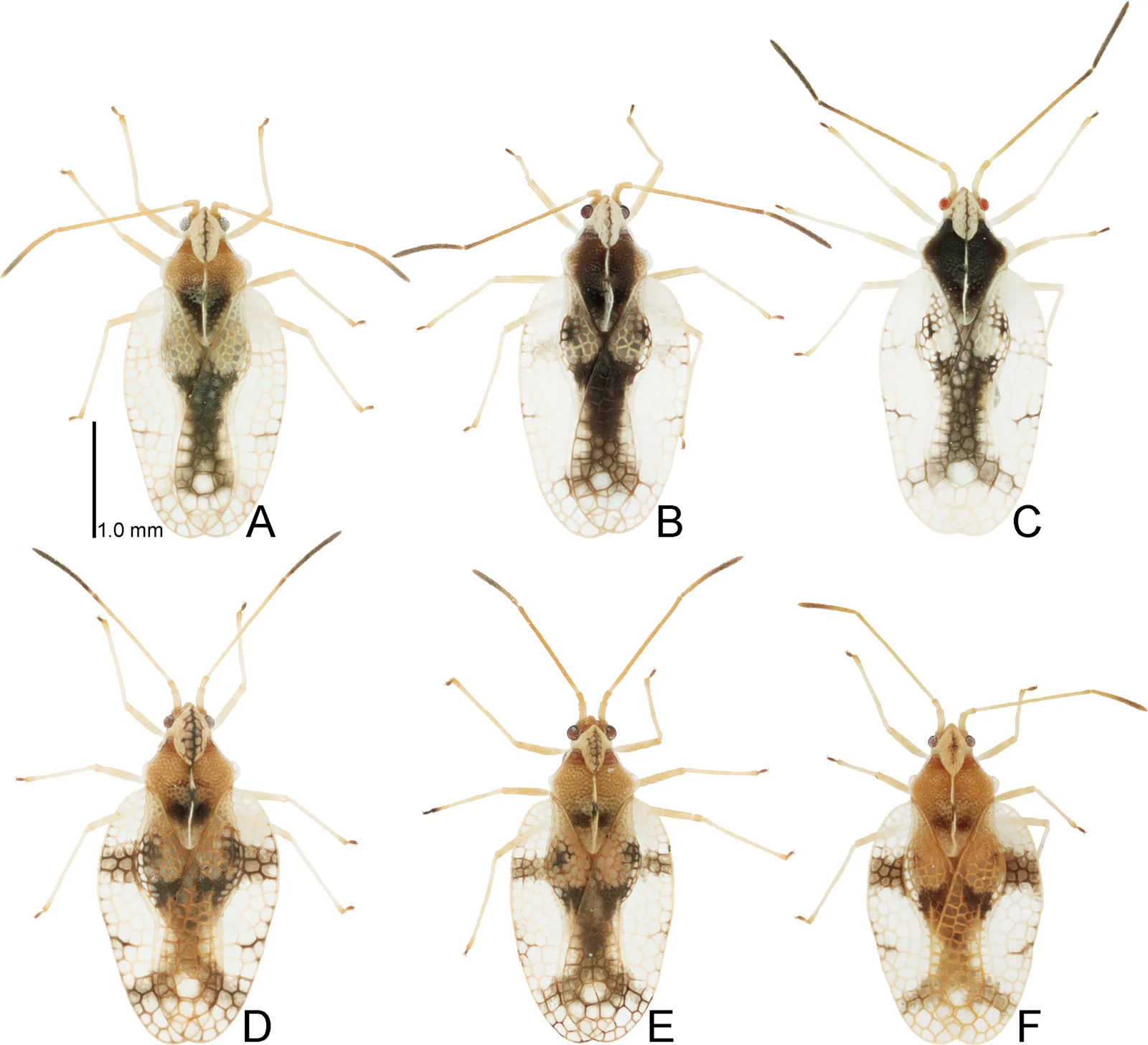
Dorsal habitus of two *Omoplax* species endemic to the Ogasawara Islands of Japan. **A–C**. *O. desecta*, male; **D**. *O. desecta*, female; **E**. *O. sandoru* sp. nov., male; **F**. *O. sandoru* sp. nov., female.

**Figure 5.**
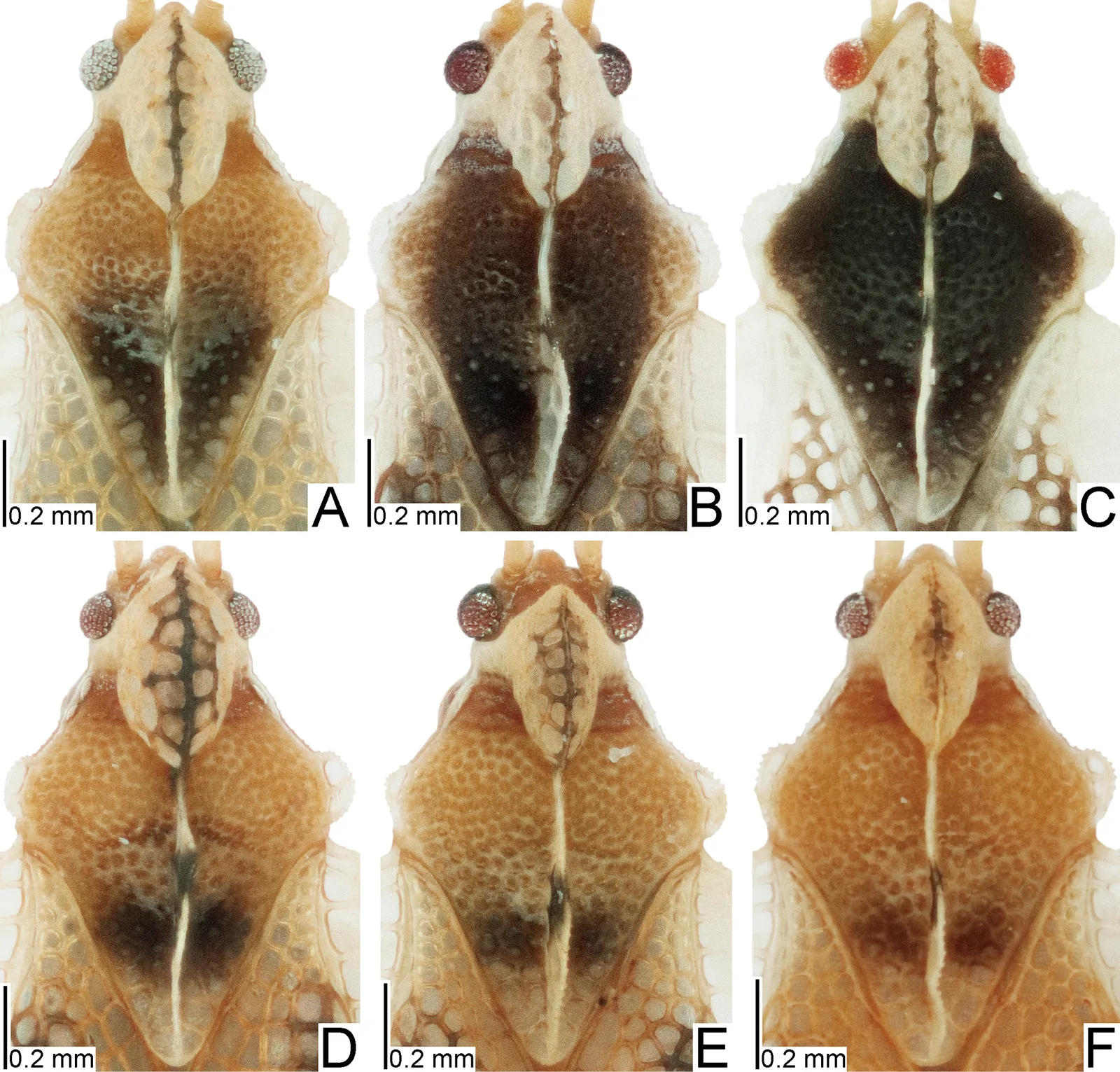
Pronota of two *Omoplax* species endemic to the Ogasawara Islands of Japan, dorsal view. **A–C**. *O. desecta*, male; **D**. *O. desecta*, female; **E**. *O. sandoru* sp. nov., male; **F**. *O. sandoru* sp. nov., female.

**Figure 6.**
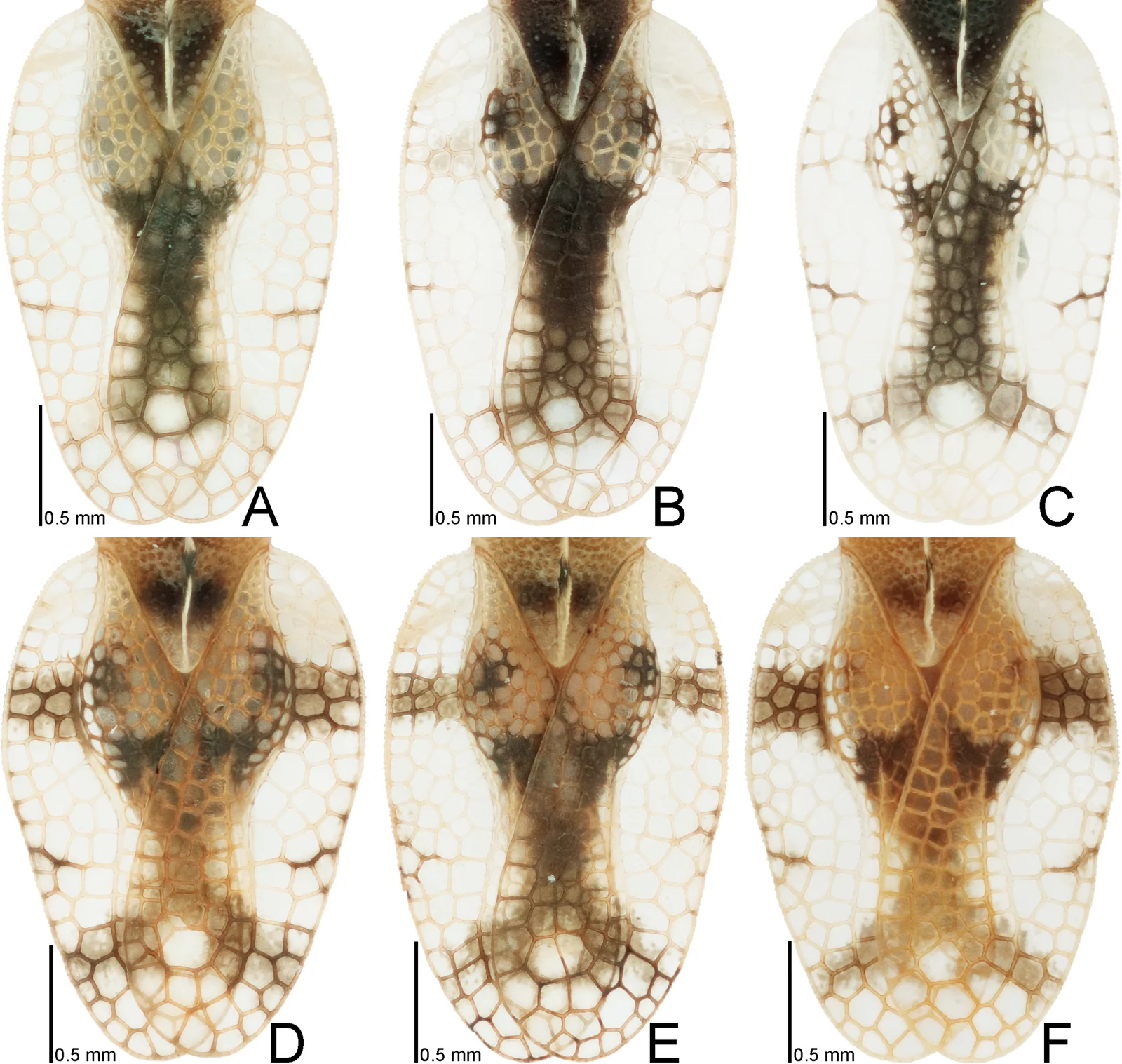
Hemelytra of two *Omoplax* species endemic to the Ogasawara Islands of Japan, dorsal view. **A–C**. *O. desecta*, male; **D**. *O. desecta*, female; **E**. *O. sandoru* sp. nov., male; **F**. *O. sandoru* sp. nov., female.

**Figure 7.**
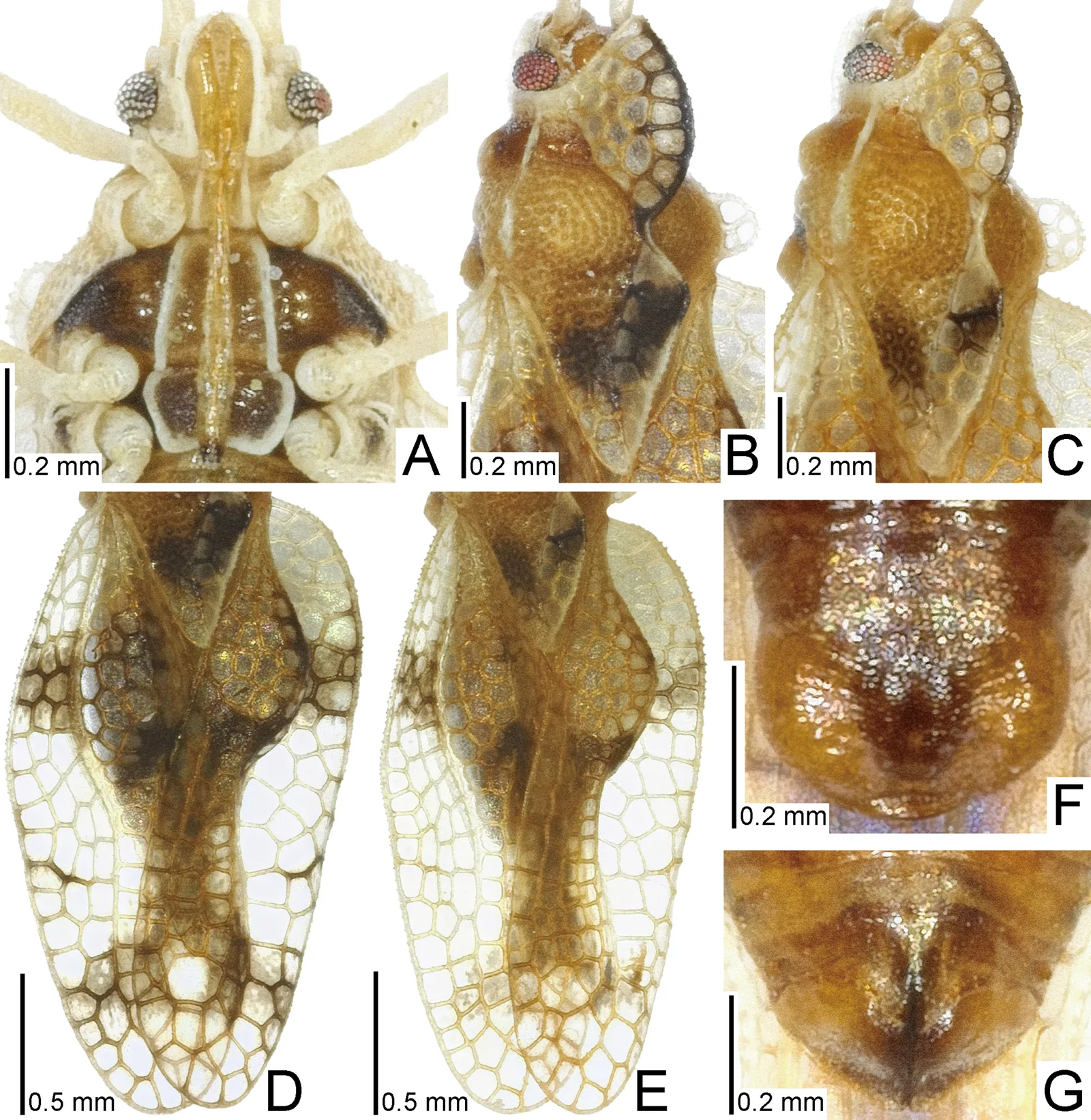
Detailed morphological images of *Omoplax sandoru* sp. nov. endemic to the Ogasawara Islands of Japan. **A**. Rostrum, ventral view; **B**. Male pronotum, dorsolateral view; **C**. Female pronotum, dorsolateral view; **D**. Male hemelytra, dorsolateral view; **E**. Female hemelytra, dorsolateral view; **F**. Male terminalia, ventral view; **G**. Female terminalia, ventral view.

**Figure 8.**
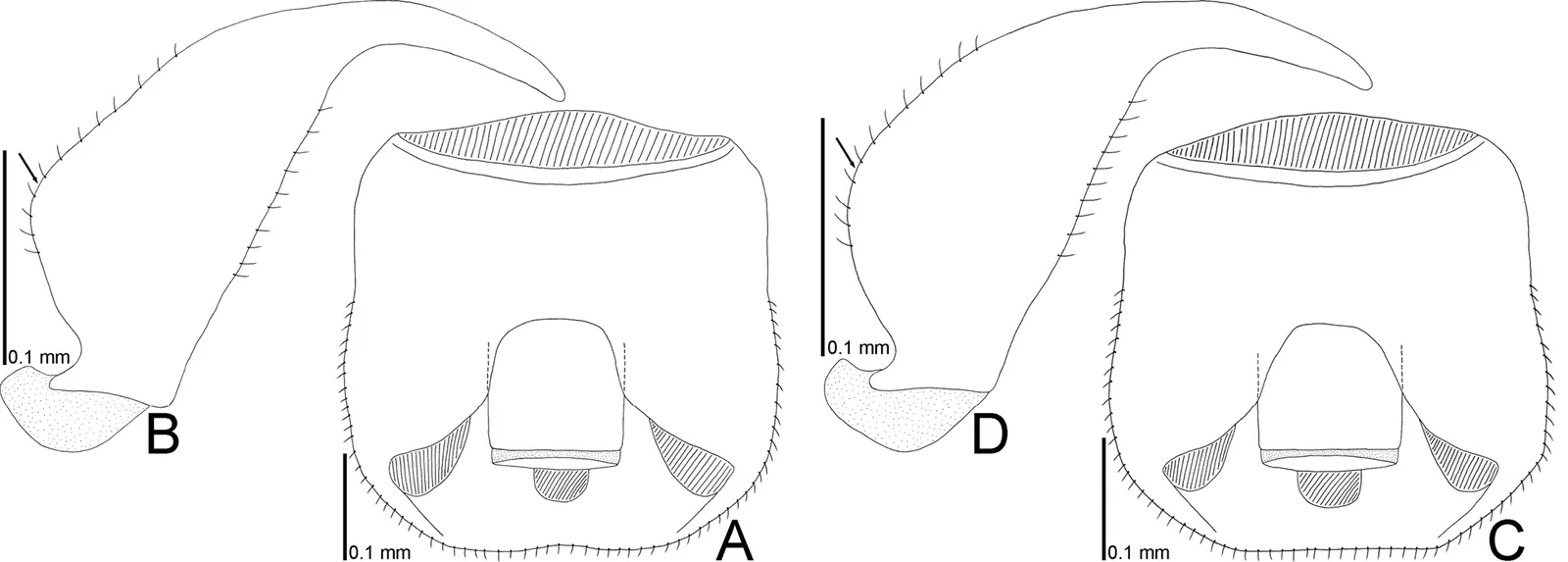
Line drawings of male genitalia of two *Omoplax* species endemic to the Ogasawara Islands of Japan, dorsal view. **A**. *O. desecta*, pygophore; **B**. *O. desecta*, paramere; **C**. *O. sandoru* sp. nov., pygophore; **D**. *O. sandoru* sp. nov., paramere. Arrows indicate important morphological differences.

The maximum likelihood (ML) tree of 4,452 SNPs well supported the monophyly of five *Omoplax* species, namely, *O. hisasuei*, *O. karubei*, *O. kobugashi*, *O. inugusu*, and *O. majorcarinae* (SH-aLRT support values = 99.9–100%; UFBoot support values = 100%) (Fig. 3). In contrast, the monophyly of *O. desecta*, as identified based on previous studies (e.g., Souma and Kamitani 2021; Souma 2022a, 2025), was not supported, and its populations in Chichijima and Hahajima groups formed monophyletic clades (SH-aLRT support values = 100%; UFBoot support values = 100%). The ML tree also supported the following phylogenetic relationships within the genus *Omoplax* (SH-aLRT support values = 96–100%; UFBoot support values = 87–100%): *O. kobugashi* + ((*O. majorcarinae* + (*O. hisasuei* + population in Hahajima Group of *O. desecta*, as identified based on previous studies)) + (*O. karubei* + population in Chichijima Group of *O. desecta*, as identified based on previous studies)). Considering the results of the Neighbor-Net, PCA, and ML tree, the populations in Chichijima and Hahajima groups of *O. desecta*, as identified based on previous studies, should be considered two distinct species. Because of morphological differences (Figs 4, 5, 6, 7, 8) and the exact island of the type locality, as detailed in the section below, the populations in Chichijima and Hahajima groups were treated as *O. desecta* and *O. sandoru* sp. nov., respectively. The monophyly of the host race feeding on *C. inophyllum* of *O. desecta* was well supported by the ML tree (SH-aLRT support value = 100%; UFBoot support value = 100%). However, *O. desecta* did not form monophyletic clades in any of the three populations on the islands of Anijima, Chichijima, or Ototojima within Chichijima Group.

### Systematics

#### Genus Omoplax

Taxon classification

Animalia

HemipteraTingidae

Horváth, 1912

213B8A77-E6FD-5ACC-B39C-2B2F6E988BA3

Omoplax Horváth, 1912: 336 (as subgenus of Stephanitis Stål, 1873; upgraded by Takeya 1962: 74). Type species by monotypy: Stephanitis (Omoplax) desecta Horváth, 1912.

##### Japanese common name.

Ogasawara-gunbai-zoku.

##### Note.

For detailed diagnostic characters of the genus, see Souma and Kamitani (2021) and Souma (2022a).

##### Remarks.

The genus *Omoplax* is endemic to the Ogasawara Islands of Japan and previously comprised the following seven species: *O. desecta*, *O. hisasuei*, *O. inugusu*, *O. karubei*, *O. kobugashi*, *O. majorcarinae*, and *O. mukojimensis* (Souma 2025). The present study describes a new species, *O. sandoru* sp. nov., which increases the total number of species to eight. The species sections below cover only *O. desecta*, *O. karubei*, and *O. sandoru* sp. nov., for which new information is presented.

#### Omoplax desecta

Taxon classification

Animalia

HemipteraTingidae

(Horváth, 1912)

B33A211F-9425-57A5-B1D2-52E3860663C3

**Figure 9.**
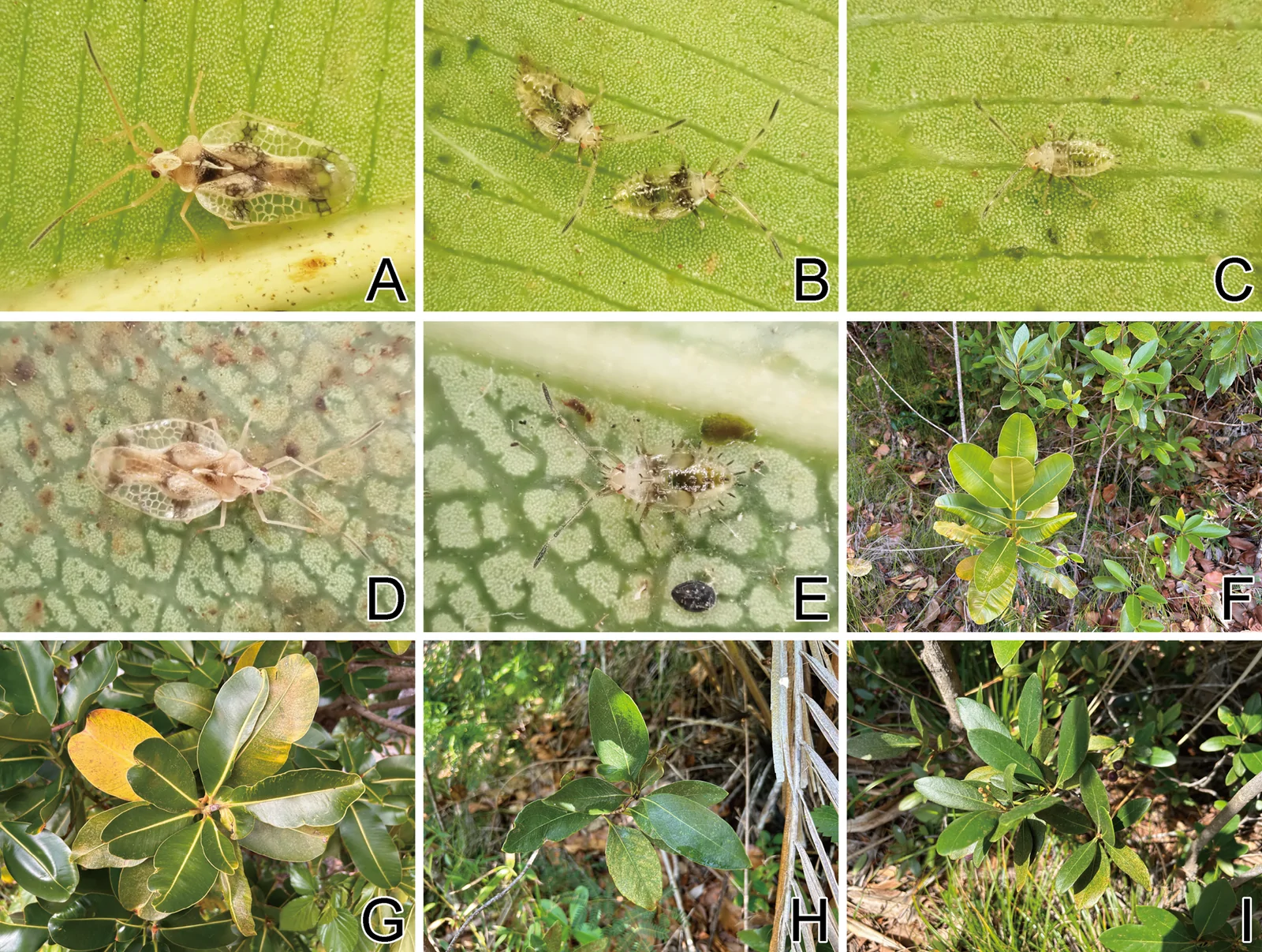
Living individuals of *Omoplax desecta* from Chichijima Island, the Ogasawara Islands, Japan. **A**. Host race feeding on *Calophyllum inophyllum*, male; **B**. Host race feeding on C. inophyllum, fifth instar nymph; **C**. Host race feeding on *C. inophyllum*, fourth instar nymph; **D**. Host race feeding on Rhaphiolepis indica var. tashiroi, female; **E**. Host race feeding on R. indica var. tashiroi, fifth instar nymph. Host plants of two *Omoplax* species endemic to the Ogasawara Islands; **F**. Adjacent *C. inophyllum* and R. indica var. tashiroi on Chichijima Island, both damaged by *O. desecta*; **G**. *C. inophyllum* on Chichijima Island, damaged by *O. desecta*; **H**. R. indica var. tashiroi on Chichijima Island, damaged by *O. desecta*; **I**. R. indica var. tashiroi on Hahajima Island, damaged by *O. sandoru* sp. nov.

Figs 4A–D, 5A–D, 6A–D, 8A, 8B, 9A–E

Stephanitis (Omoplax) desecta Horváth, 1912: 337. Syntype(s): ♀; type locality: Japan • Ogasawara [= Ogasawara Islands, Chichijima Group, Chichijima Island]; Laboratory of Systematic Entomology, Faculty of Agriculture, Hokkaido University, Sapporo, Japan (cf. Matsumura 1906: 13; Tomokuni 1994: 844; Souma 2022a: 125).
Omoplax desecta : Takeya (1962: 74) (new combination).

##### Japanese common name.

Ogasawara-gunbai.

##### Note.

For detailed diagnostic characters and other references, see Souma (2025) and the identification key below.

##### Material examined.

Japan • 2 fifth instar nymphs; Ogasawara Isls, Chichijima Is., Higashi-machi; *Calophyllum inophyllum*; 21 Apr. 2021; Y. Nakatani leg.; DDBJ accession numbers SAMN60765439–SAMN60765440; SIHU • 3 ♂♂ 5 ♀♀; same locality and host plant data as for preceding; 8 Oct. 2024; J. Souma leg.; DDBJ accession numbers SAMN60765441–SAMN60765448; SIHU • 1 ♀; same locality and host plant data as for preceding; 7 Mar. 2026; J. Souma leg.; SIHU • 5 ♂♂ 5 ♀♀; Ogasawara Isls, Anijima Is., Tamana Beach–Mt. Mikaeri; Rhaphiolepis indica var. tashiroi; 24 Sep. 2024; J. Souma leg.; DDBJ accession numbers SAMN60765480–SAMN60765489; SIHU • 1 ♀; Ogasawara Isls., Chichijima Is., Mt. Mikazuki; Rhaphiolepis indica var. tashiroi; 10 Jan. 2025; Y. Hisasue leg.; DDBJ accession number SAMN60765469; SIHU • 5 ♂♂ 5 ♀♀; Ogasawara Isls, Ototojima Is., Hironesawa; Rhaphiolepis indica var. tashiroi; 5 Jul. 2025; Y. Hisasue leg.; DDBJ accession numbers SAMN60765457–SAMN60765466; SIHU • 3 ♀♀; Ogasawara Isls, Anijima Is., Futamata Cape; *Calophyllum inophyllum*; 12 Oct. 2025; Y. Hisasue leg.; SIHU • 1 ♀; Ogasawara Isls., Chichijima Is., Shigure Dam; Rhaphiolepis indica var. tashiroi; 7 Mar. 2026; J. Souma leg.; SIHU • 1 ♀; Ogasawara Isls, Chichijima Is., Mt. One; *Calophyllum inophyllum*; 8 Mar. 2026; J. Souma leg.; SIHU • 1 ♂; Ogasawara Isls, Chichijima Is., Oku-mura; *Calophyllum inophyllum*; 8 Mar. 2026; J. Souma leg.; SIHU • 1 ♂; Ogasawara Isls, Chichijima Is., Miyanohama; *Calophyllum inophyllum*; 9 Mar. 2026; J. Souma leg.; SIHU • 1 ♂ 1 ♀; Ogasawara Isls, Chichijima Is., Ogamiyama Park; *Calophyllum inophyllum*; 9 Mar. 2026; J. Souma leg.; SIHU • 1 ♀; same locality, date, and collector data as for preceding; Rhaphiolepis indica var. tashiroi; SIHU • 2 ♂♂ 1 ♀; Ogasawara Isls, Chichijima Is., Byobu Valley; *Calophyllum inophyllum*; 10 Mar. 2026; J. Souma leg.; SIHU • 1 ♂; Ogasawara Isls, Chichijima Is., Fukiage Valley; *Calophyllum inophyllum*; 10 Mar. 2026; J. Souma leg.; SIHU • 1 ♂; Ogasawara Isls, Chichijima Is., Kopepe Beach; *Calophyllum inophyllum*; 10 Mar. 2026; J. Souma leg.; SIHU • 1 ♀; Ogasawara Isls, Chichijima Is., Kominato; *Calophyllum inophyllum*; 10 Mar. 2026; J. Souma leg.; SIHU • 1 ♂; Ogasawara Isls., Chichijima Is., Mt. Kuwanoki; Rhaphiolepis indica var. tashiroi; 10 Mar. 2026; J. Souma leg.; SIHU • 3 ♂♂ 1 ♀; Ogasawara Isls., Ototojima Is., Kurohama–Ichinotani; Rhaphiolepis indica var. tashiroi; 12 Mar. 2026; J. Souma leg.; SIHU • 1 ♀; Ogasawara Isls., Chichijima Is., Mt. Asahi; Rhaphiolepis indica var. tashiroi; 13 Mar. 2026; J. Souma leg.; SIHU • 1 ♂ 1 ♀; Ogasawara Isls., Chichijima Is., Mt. Noryo; Rhaphiolepis indica var. tashiroi; 13 Mar. 2026; J. Souma leg.; SIHU • 1 ♀; Ogasawara Isls, Chichijima Is., Hatsuneura; *Calophyllum inophyllum*; 15 Mar. 2026; J. Souma leg.; SIHU • 1 ♂; same locality, date, and collector data as for preceding; Rhaphiolepis indica var. tashiroi; SIHU • 2 ♂♂ 1 ♀; Ogasawara Isls, Chichijima Is., Kiyose; *Calophyllum inophyllum*; 16 Mar. 2026; J. Souma leg.; SIHU • 1 ♂; Ogasawara Isls, Chichijima Is., Nishi-machi; *Calophyllum inophyllum*; 16 Mar. 2026; J. Souma leg.; SIHU. The two nymphs recorded above are in poor condition and are not described in the present study.

##### Remarks.

In the original description (Horváth 1912), the type locality of *Omoplax desecta* is given as “Ogasawara” [= Ogasawara Islands], meaning the name of the island where the syntype was collected is unknown. Although the label on the syntype contains no additional information regarding the type locality, the date and collector are given as “20 VIII 1905” and “Matsumra” [= Shonen Matsumura], respectively (Tomokuni 1994; Souma 2022a: fig. 7). During his 1905 survey of the Ogasawara Islands, the late Dr. Shonen Matsumura visited only Chichijima Island (Matsumura 1906). Consequently, the exact island of the type locality of *O. desecta* is Chichijima Island, Chichijima Group, the Ogasawara Islands.

Based on molecular phylogenetic analysis and morphological observations, the populations in Chichijima and Hahajima groups, which were identified as *O. desecta* (e.g., Souma and Kamitani 2021; Souma 2022a, 2025), should be considered as two distinct species. In accordance with the exact island of the type locality, the population in Chichijima Group was identified as *O. desecta*, and the population in Hahajima Group was described as *O. sandoru* sp. nov. in the section below. Conversely, the previous record of *O. desecta* in Mukojima Group (Souma 2022a) was based on a single female specimen missing the left hemelytron from Nakodojima Island and is provisionally retained as *O. desecta* in the present study.

There are two host races of *O. desecta*, one that feeds on *Calophyllum inophyllum* (Fig. 9A–C) and another on Rhaphiolepis indica var. tashiroi (Fig. 9D, E). The former host race is common in urban areas where *C. inophyllum* is planted and in coastal areas. The latter host race is common in mountainous areas. However, in the mountains bordering the sea, both host races occasionally occur sympatrically (Fig. 9F). Although weak genetic differences were confirmed (Fig. 1), the two host races are considered a single species due to the absence of morphological differences.

##### Distribution.

**Figure 10.**
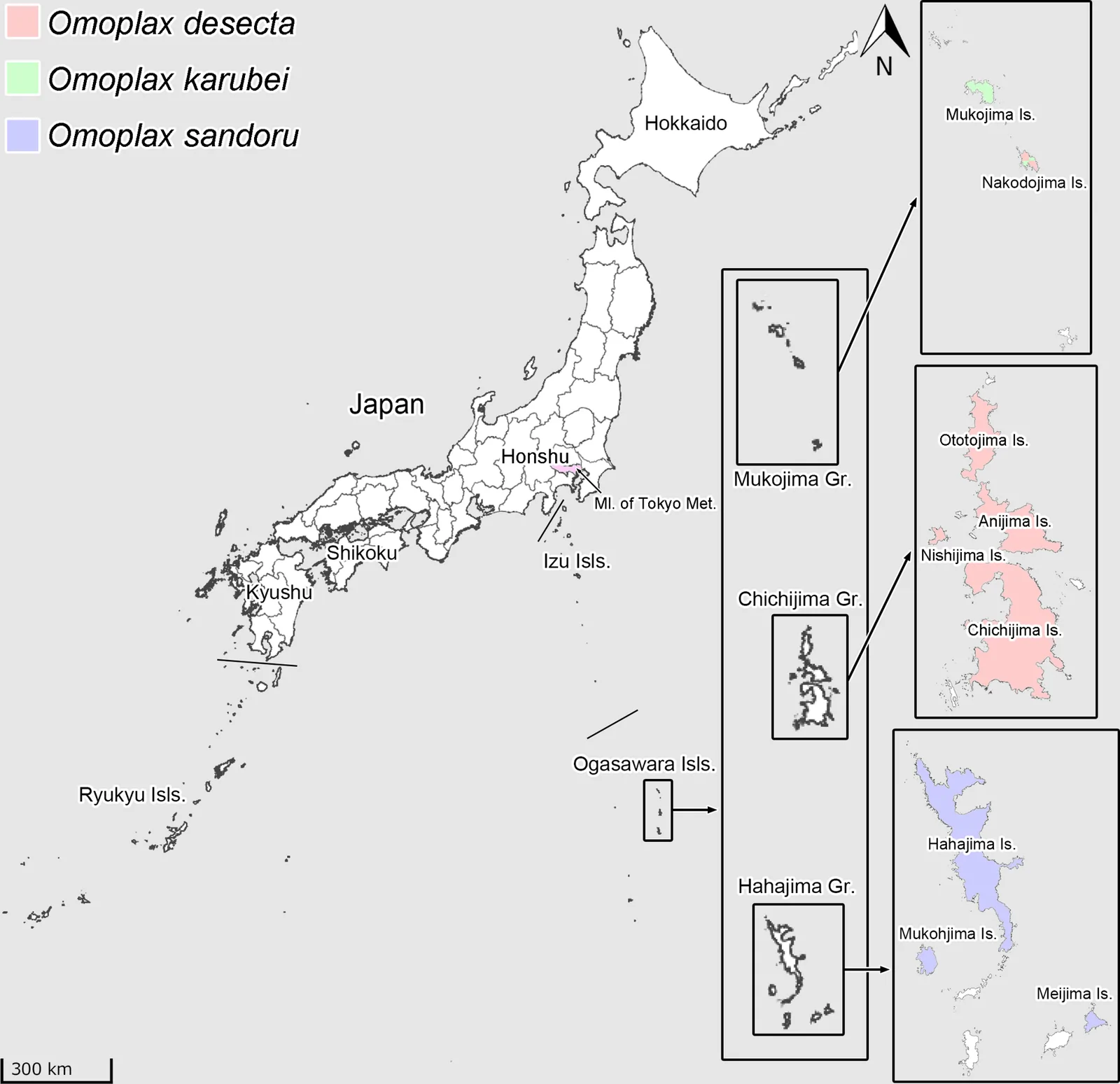
Distribution ranges of three *Omoplax* species endemic to the Ogasawara Islands of Japan. The distances between the main regions on the map are as follows: mainland of the Tokyo Metropolis and the Mukojima Islands, approximately 900 km; Mukojima and Chichijima groups, approximately 30 km; and Chichijima and Hahajima groups, approximately 40 km. No lace bug has been recorded on any of the uncolored islands in the Ogasawara Islands. The colored islands within the same island group are all less than 10 km apart. The map was created using data from the Geospatial Information Authority of Japan (GSI) (https://maps.gsi.go.jp).

Japan: Ogasawara Islands: Chichijima Group (Anijima Island, Chichijima Island, Nishijima Island, Ototojima Island), Mukojima Group (Nakodojima Island) (Fig. 10; Suppl. material 2).

*Omoplax desecta* is endemic to Chichijima and Mukojima groups. Molecular phylogenetic analysis and morphological observations revealed that the populations in Chichijima and Hahajima groups, which were identified as *O. desecta* (e.g., Souma and Kamitani 2021; Souma 2022a, 2025), should be considered as two distinct species. Based on the exact island of the type locality mentioned above, the population in Chichijima Group corresponds to *O. desecta*. Therefore, all previous records of *O. desecta* from the Hahajima Group (Nakane 1970; Yasunaga et al. 1993; Miyano 1998; Guilbert 2001; Government of Japan 2010; Souma and Kamitani 2021; Souma 2022a, 2025) should be corrected to *O. sandoru* sp. nov., which was recognized as a population in Hahajima Group of *O. desecta*.

To the best of our knowledge, the host race of *O. desecta* feeding on *Calophyllum inophyllum* has been observed on the islands of Anijima and Chichijima. The host race *O. desecta* feeding on Rhaphiolepis indica var. tashiroi occurs on the islands of Anijima, Chichijima, Nishijima, and Ototojima (cf. Souma 2025). No information is available regarding the host race on Nakodojima Island (cf. Souma 2022a).

##### Host plant.

*Calophyllum inophyllum* (Malpighiales, Calophyllaceae) (Fig. 9G) and Rhaphiolepis indica var. tashiroi (Rosales, Rosaceae) (Fig. 9H) (Souma 2025).

*Omoplax desecta* feeds on only two species of evergreen broad-leaved trees belonging to different orders. Host selection of this species should be considered as an example of extended host specificity rather than generalism (cf. Kato and Imada 2025).

##### Bionomics.

*Omoplax desecta* inhabits an evergreen broad-leaved forest with a subtropical climate in Chichijima and Mukojima groups and sucks sap from the abaxial side of the leaves of *Calophyllum inophyllum* and Rhaphiolepis indica var. tashiroi, causing irregular yellowing on the adaxial side (Souma 2025).

Adults were collected during all seasons; nymphs were collected in March, April, and September (Drake 1956; Nishimura and Arai 1989; Tomokuni 1994; Guilbert 2001; Souma and Kamitani 2021; Souma 2022a, 2025).

On Anijima Island, *O. desecta* has been confirmed in the stomach contents of the invasive green anole *Anolis carolinensis* (Squamata, Dactyloidae) (Takahashi et al. 2014).

#### Omoplax karubei

Taxon classification

Animalia

HemipteraTingidae

Souma, 2022

C090738F-8395-505F-899A-6BA931F543D4

Omoplax karubei Souma, 2022a: 118. Holotype: ♂; type locality: Japan • “東京都小笠原村, 聟島” [= Ogasawara Islands, Mukojima Group, Mukojima Island]; Kanagawa Prefectural Museum of Natural History, Kanagawa, Japan.

##### Japanese common name.

Karube-gunbai.

##### Note.

For detailed diagnostic characters and other references, see Souma (2025) and the identification key below.

##### Material examined.

Japan • 1 ♂ 1 ♀; Ogasawara Isls, Nakodojima Is.; Rhaphiolepis indica var. tashiroi; 10 Mar. 2026; Y. Hisasue leg.; SIHU.

##### Distribution.

Japan: Ogasawara Islands: Mukojima Group (Mukojima Island, Nakodojima Island) (Fig. 10; Suppl. material 2).

*Omoplax karubei* is newly recorded from Nakodojima Island and is endemic to Mukojima Group.

##### Host plant.

Rhaphiolepis indica var. tashiroi (Souma 2025).

##### Bionomics.

*Omoplax karubei* inhabits an evergreen broad-leaved forest with a subtropical climate in the Ogasawara Islands and sucks sap from the abaxial side of the leaves of Rhaphiolepis indica var. tashiroi, causing irregular yellowing on the adaxial side (Souma 2025).

Adults were collected in March, June, and July; nymphs are unknown (Souma 2022a, 2025).

#### Omoplax sandoru

Taxon classification

Animalia

HemipteraTingidae

Souma sp. nov.

5EF97720-8E85-5F17-B85C-1FF03839644B

https://zoobank.org/A19E0A1E-02A5-449D-9431-4DE337E2126E

Figs 4E, 4F, 5E, 5F, 6E, 6F, 7A–G, 8C, 8D

Omoplax desecta (Horváth, 1912): Nakane (1970: 28) (checklist); Yasunaga et al. (1993: 179) (monograph: part); Miyano (1998: 8) (distribution); Guilbert (2001: 551) (distribution: part); Government of Japan (2010: 208) (checklist: part); Yamada and Tomokuni (2012: 198) (monograph: part); Yamada and Ishikawa (2016: 432) (checklist: part); Souma and Kamitani (2021: 8) (distribution: part); Souma (2022a: 125) (distribution: part); Shimamoto and Ishikawa (2023: 93) (catalog: part); Souma (2025: 249) (distribution: part). Misidentifications.

##### Japanese common name.

Sandoru-gunbai.

##### Type material.

***Holotype***, Japan • ♂; Ogasawara Isls, Hahajima Is., Mt. Chibusa; Rhaphiolepis indica var. tashiroi; 2 Oct. 2024; J. Souma; SIHU. ***Paratypes***, Japan • 1 ♂; same data as for holotype; SIHU • 18 ♂♂; Ogasawara Isls, Hahajima Is., Minamizaki; 19 Apr. 1997; K. Matsumoto leg; NSMT • 1 ♂; same locality data as for preceding; 4 Jul. 1997; T. Kishimoto leg; NSMT • 1 ♂; same locality data as for preceding; 15 Jul. 2024; Y. Uehara leg.; SIHU • 4 ♂♂ 6 ♀♀; same locality data as for preceding; Rhaphiolepis indica var. tashiroi; 28 Sep. 2024; J. Souma leg.; DDBJ accession numbers SAMN60765470–SAMN60765479; SIHU • 3 ♂♂; same locality data as for preceding; 3 Sep. 2026; Y. Hisasue leg.; SIHU • 1 ♂; Ogasawara Isls, Hahajima Is., Nakanotaira–Minamizaki; 19 Apr. 1997; K. Matsumoto leg; NSMT • 1 ♂; Ogasawara Isls, Hahajima Is., Mt. Mikazuki (non-existent place name: mislabeling?); 19 Apr. 1997; K. Matsumoto leg; NSMT • 1 ♂; Ogasawara Isls, Hahajima Is., Komori Valley; 20 Apr. 1997; K. Matsumoto leg; NSMT • 1 ♂; Ogasawara Isls, Hahajima Is., Mt. Yakeyama; 20 Apr. 1997; K. Matsumoto leg; NSMT • 3 ♂♂; Ogasawara Isls, Hahajima Is., Oki-mura; 20 Apr. 1997; K. Matsumoto leg; NSMT • 2 ♂♂; Ogasawara Isls, Hahajima Is., Nishiura; 4 Jul. 1997; K. Matsumoto leg; NSMT • 1 ♂; Ogasawara Isls, Hahajima Is., Tamagawa Dam; 5 Jul. 1997; K. Matsumoto leg.; NSMT • 1 ♂; Ogasawara Isls, Hahajima Is., Igumadani, Sekimon; alt. 370 m; 26 May 2022; T. Yoshida leg.; SIHU • 2 ♂♂; Ogasawara Isls, Meijima Is.; 14 Jun. 2024; N. Tsuji leg.; SIHU • 1 ♂; Ogasawara Isls, Hahajima Is., Nishidai; 17 Jun. 2024; N. Tsuji leg.; SIHU • 1 ♂; Ogasawara Isls, Hahajima Is., Shizukazawa; 20 Jun. 2024; Y. Hisasue leg.; SIHU • 5 ♂♂; Ogasawara Isls, Mukohjima Is.; alt. 0–90 m; 16 Jul. 2024; Y. Uehara leg.; SIHU • 1 ♂; Ogasawara Isls, Hahajima Is., Shin-yuhigaoka; 6 Sep. 2026; Y. Hisasue leg.; SIHU. All specimens collected until 2025 without DDBJ accession numbers were recorded as *Omoplax desecta* by Souma (2025).

##### Additional material examined.

Japan • 5 ♀♀; same data as for holotype; SIHU • 1 ♀; same locality data as for holotype; 8 Jun. 1976; T. Nakane leg.; NSMT • 1 ♀; same locality data as for holotype; 6 Jul. 1997; T. Kishimoto leg; NSMT • 2 ♀♀; same locality data as for holotype; 7 Jul. 1997; K. Matsumoto leg; NSMT • 1 ♀; same locality data as for holotype; 27 May 2022; T. Yoshida leg.; SIHU • 1 ♀; Ogasawara Isls, Hahajima Is., Higashiko; 5 Jun. 1976; T. Nakane leg.; NSMT • 2 ♀♀; Ogasawara Isls, Hahajima Is., Kuwanoki-zawa; 4 Aug. 1996; K. Matsumoto leg.; NSMT • 14 ♀♀; Ogasawara Isls, Hahajima Is., Minamizaki; 19 Apr. 1997; K. Matsumoto leg; NSMT • 1 ♀; same locality data as for preceding; Rhaphiolepis indica var. tashiroi; 27 Sep. 2024; J. Souma leg.; SIHU • 1 ♀; same locality data as for preceding; 3 Sep. 2026; Y. Hisasue leg.; SIHU • 3 ♀♀; Ogasawara Isls, Hahajima Is., Nakanotaira–Minamizaki; 19 Apr. 1997; K. Matsumoto leg; NSMT • 1 ♀; Ogasawara Isls, Hahajima Is., Komori Valley; 20 Apr. 1997; K. Matsumoto leg; NSMT • 1 ♀; Ogasawara Isls, Hahajima Is., Mt. Yakeyama; 20 Apr. 1997; K. Matsumoto leg; NSMT • 1 ♀; Ogasawara Isls, Hahajima Is., Oki-mura; 20 Apr. 1997; T. Kishimoto leg; NSMT • 1 ♀; Ogasawara Isls, Hahajima Is., Mt. Kuwanoki; 4 Jul. 1997; T. Kishimoto leg; NSMT • 1 ♀; Ogasawara Isls, Hahajima Is., Tamagawa Dam; 5 Jul. 1997; K. Matsumoto leg.; NSMT • 1 ♀; same locality data as for preceding; 14 Jul. 2024; Y. Uehara leg.; SIHU • 1 ♀; Ogasawara Isls, Meijima Is.; 14 Jun. 2024; N. Tsuji leg.; SIHU • 5 ♀♀; Ogasawara Isls, Hahajima Is., Shizukazawa; 20 Jun. 2024; Y. Hisasue leg.; SIHU • 1 ♀; Ogasawara Isls, Hahajima Is., Nijuccho Pass; alt. 160 m; 15 Jul. 2024; Y. Uehara leg.; SIHU • 1 ♀; Ogasawara Isls, Mukohjima Is.; alt. 0–90 m; 16 Jul. 2024; Y. Uehara leg.; SIHU • 1 ♀; Ogasawara Isls, Hahajima Is., Hyogidaira; Rhaphiolepis indica var. tashiroi; 9 Mar. 2025; J. Souma leg.; SIHU • 1 ♀; same locality, host plant, and collector data as for preceding; 11 Mar. 2025; SIHU • 1 ♀; Ogasawara Isls, Hahajima Is., Nishiura; 4 May 2025; Y. Hisasue leg.; SIHU • 1 ♀; Ogasawara Isls, Hahajima Is., Shin-yuhigaoka; 6 Sep. 2026; Y. Hisasue leg.; SIHU. All specimens collected until 2025 were previously recorded as *Omoplax desecta* (Souma 2025) but were identified as a new species in this study based on the morphological characteristics of the female paratypes used for MIG-seq analysis.

##### Diagnosis.

*Omoplax sandoru* sp. nov. can be separated from the other *Omoplax* species based on a combination of the following characteristics: antennal segment III in apex pale brown (Fig. 4E, F); rostrum reaching posterior margin of metasternum (Fig. 7A); pronotal disc pale brown (Fig. 5E, F); hood > 0.5× as wide as maximum width of head across compound eyes, not reaching apex of clypeus; paranotum without areolae in middle part, with areolae in remaining parts (Fig. 7B, C); anterior margin of hemelytron weakly curved downward in apical half (Fig. 7D, E); subcostal and discoidal areas of hemelytron not united; costal area narrower than combined width of subcostal and discoidal areas (Fig. 6E, F); Sc (subcosta) vein of hemelytron distinct in apical part of dorsal view; R+M (fused radius and media) vein of hemelytron indistinct, not carinate; ventral surface of body brown (Fig. 7F, G); and outer margin of paramere in middle part roundly convex (Fig. 8D).

##### Description.

***Male***. Head, calli, legs, and ventral surface of body in various shades of brown; antennal segments I–III, pronotal disc, hood, median carina of pronotum, paranotum, posterior process, and hemelytron pale brown; antennal segment IV and markings on dorsum dark brown to black; compound eye dark red; areolae of pronotum and hemelytron transparent; pubescence on body yellowish (Figs 4E, 5E, 6E, 7A, 7B, 7D, 7F).

Body ovate; pubescence on body shorter than radius of compound eye (Figs 4E, 7F). Head (Figs 5E, 7B) glabrous; pair of frontal spines separated from each other at apices, not reaching apex of clypeus, occasionally reduced; median spine not reaching bases of frontal spines, occasionally reduced; pair of occipital spines not reaching anterior margin of compound eyes, occasionally reduced; antenniferous tubercle obtuse, curved inward, longer than frontal spines; vertex and clypeus smooth. Compound eye round in dorsal view. Antenna densely covered with minute pubescence on segments I–III and long pubescence on segment IV; pubescence on segment IV longer than pubescence on other parts of body; segment I cylindrical, shorter than segment IV; segment II cylindrical, shortest among antennal segments; segment III linear, longest amongst antennal segments; segment IV fusiform. Bucculae closed at anterior ends, with 3 rows of areolae at highest part. Rostrum (Fig. 7A) reaching posterior margin of metasternum.

Pronotum (Figs 5E, 7B) glabrous. Pronotal disc coarsely punctate. Hood shorter than median carina of pronotum, higher than median carina, with 3–5 rows of areolae at highest part, > 0.5× as wide as maximum width of head across compound eyes, not reaching apex of clypeus, without robust denticles throughout its length; dorsal margin arched; posterior margin extending to anterior part of pronotal disc. Collar not covering compound eye. Median carina straight, extending to apex of posterior process, with 1–2 rows of areolae at highest part, without robust denticles throughout its length; dorsal margin arched. Calli smooth. Paranotum subvertical, widened posteriad, with a single row of areolae in anterior part and 1–2 rows in posterior part, without areolae in middle part; outer margin gently curved outward in posterior part and straight in remaining parts, without robust denticles throughout its length. Posterior process triangular; apex rounded.

Hemelytron (Figs 6E, 7D) glabrous, extending beyond apex of abdomen; anterior margin weakly curved downward in apical half; apices close to each other at rest; subcostal and discoidal areas not united; costal area narrower than combined width of subcostal and discoidal areas, with 4–5 rows of areolae at widest part; subcostal area with 3 rows of areolae at widest part; discoidal area with 4 rows of areolae at widest part; sutural area with 4–5 rows of areolae at widest part; hypocostal lamina with a single row of areolae throughout its length; Sc (subcostal) and Hc (hypocosta) veins distinct throughout their respective length; R+M (fused radius and media) and Cu (cubitus) veins indistinct throughout their respective length, not carinate; Sc and R+M veins without robust denticles throughout their respective length; Sc vein distinct in apical part of dorsal view.

Thoracic pleura smooth in anterior part, coarsely punctate in posterior part. Ostiolar peritreme oblong. Sternal laminae (Fig. 7A) lower than bucculae; pro- and mesosternal laminae open at both anterior and posterior ends; metasternal laminae as high as mesosternal laminae, open at anterior ends, fused with each other at posterior ends. Legs (Fig. 4E) smooth, covered with pubescence; femora thickest at middle. Abdomen ellipsoidal. Pygophore (Figs 7F, 8C) compressed dorsoventrally, semicircular in ventral view, covered with minute pubescence; anterior margin not concave in middle part. Paramere (Fig. 8D) sickle-like, expanded in middle part, covered with minute pubescence in middle part of outer and inner margins; outer margin roundly convex in middle part.

Measurements (*n* = 50). Body length with hemelytra 2.60–2.95 mm; maximum width of body across hemelytra 1.30–1.40 mm; length of antennal segments I–IV 0.20 mm, 0.10 mm, 1.05–1.10 mm, and 0.65–0.70 mm, respectively; pronotal length 1.00–1.15 mm; pronotal width across paranota 0.70–0.75 mm; hemelytral length 1.95–2.20 mm; maximum width of hemelytron 0.80–0.90 mm.

**Female**. General habitus very similar to that of male (Figs 4F, 5F, 6F, 7C, 7E, 7G) except for the following characters: body usually longer and wider than in male; antennal segments III–IV shorter than in male; hemelytron usually longer and wider than in male; and apical part of abdomen pentagonal in ventral view.

Measurements (*n* = 55). Body length with hemelytra 2.80–3.05 mm; maximum width of body across hemelytra 1.50–1.70 mm; length of antennal segments I–IV 0.20 mm, 0.10 mm, 0.95–1.00 mm, and 0.55–0.60 mm, respectively; pronotal length 1.10–1.20 mm; pronotal width across paranota 0.75–0.80 mm; hemelytral length 2.15–2.40 mm; maximum width of hemelytron 0.85–0.95 mm.

##### Remarks.

Among all the species of *Omoplax*, *O. sandoru* sp. nov. most resembles *O. desecta* in terms of its general appearance. However, based on a comparison of the type material of the new species together with the syntype/non-type material and the original descriptions (Horváth 1912; Souma 2022a: fig. 7) of *O. desecta*, the shape of the paramere was recognized to easily differentiate *O. sandoru* sp. nov. from *O. desecta* (Fig. 8B, D): the middle part of outer margin is roundly convex in *O. sandoru* sp. nov., but angularly convex in *O. desecta*. On the other hand, the apex of antennal segment III and the pronotal disc are always pale brown in *O. sandoru* sp. nov., but sometimes dark brown to black in *O. desecta* (Figs 4, 5). The morphological differences between *O. sandoru* sp. nov. and the other seven *Omoplax* species are presented in the identification key below.

The pygophore and paramere illustrated as *O. desecta* in a previous study (Souma and Kamitani 2021) corresponded to the new species.

##### Distribution.

Japan: Ogasawara Islands: Hahajima Group (Hahajima Island, Meijima Island, Mukohjima Island) (Fig. 10; Suppl. material 2).

*Omoplax sandoru* sp. nov. is endemic to Hahajima Group.

##### Etymology.

The specific epithet is the Ogasawaran plant name “Arekisandoru” and “Sandorunoki” [= Rhaphiolepis indica var. tashiroi], referring to the host plant of the new species; a noun in apposition.

##### Host plant.

Only Rhaphiolepis indica var. tashiroi (Fig. 9I), which is also known as “Shimasharinbai” in Japanese, was confirmed as a host plant for *Omoplax sandoru* sp. nov. through field and captive observations of adults, suggesting the possibility of monophagy for this lace bug species.

##### Bionomics.

*Omoplax sandoru* sp. nov. inhabits an evergreen broad-leaved forest with a subtropical climate in Hahajima Group and sucks sap from the abaxial side of the leaves of Rhaphiolepis indica var. tashiroi, causing irregular yellowing on the adaxial side (Fig. 9I).

Adults were collected between March and November (Yasunaga et al. 1993; Miyano 1998; Guilbert 2001; Souma and Kamitani 2021; Souma 2022a, 2025); nymphs are unknown.

### Key to the species of Tingidae occurring in the Ogasawara Islands, Japan

Modified after the key provided by Souma (2025).

**Table:** 

1	Head with long spines (Souma 2025: figs 3A, 4A, 5A, 6A, 14A); paranotum narrowed posteriad; medial part of hood, median carina of pronotum, outer margin of paranotum, Sc (subcosta) and R+M (fused radius and media) veins of hemelytron with robust denticles throughout their respective length (Souma 2025: figs 7A, 8A, 9A, 10A)	***Acanthomoplax tomokunii* Souma & Kamitani, 2021**
–	Head with very short spines (Souma 2025: figs 3B–G, 4B–H, 5B–G, 6B–H, 14B–H); paranotum widened posteriad; hood, median carina of pronotum, outer margin of paranotum, Sc and R+M veins of hemelytron without robust denticles throughout their respective length (Souma 2025: figs 7B–G, 8B–H, 9B–G, 10B–H)	**2**
2	Rostrum reaching posterior margin of metasternum (Souma 2025: fig. 11B, E); ventral surface of body brown (Souma 2025: figs 12B, E, 13B, E); feeding on Rhaphiolepis indica var. tashiroi (Rosaceae) or *Calophyllum inophyllum* (Calophyllaceae)	**3**
–	Rostrum not reaching posterior margin of metasternum (Souma 2025: fig. 11C, D, F–H); ventral surface of body black (Souma 2025: figs 12C, D, F, G, 13C, D, F–H); feeding on Lauraceae	**5**
3	Hood reaching beyond apex of clypeus (Souma 2025: figs 3E, 4E, 5E, 6E, 14E); paranotum with areolae throughout its length; anterior margin of hemelytron not curved downward in apical half (Souma 2025: figs 7E, 8E, 9E, 10E); R+M vein of hemelytron distinct, carinate	***Omoplax karubei* Souma, 2022**
–	Hood not reaching apex of clypeus (Souma 2025: figs 3B, 4B, 5B, 6B, 14B); paranotum without areolae in middle part, with areolae in remaining parts; anterior margin of hemelytron weakly curved downward in apical half (Souma 2025: figs 7B, 8B, 9B, 10B); R+M vein of hemelytron indistinct, not carinate	**4**
4	Outer margin of paramere in middle part angularly convex (Fig. 8B); endemic to Mukojima and Chichijima groups	***O. desecta* (Horváth, 1912)**
–	Outer margin of paramere in middle part roundly convex (Fig. 8D); endemic to Hahajima Group	***O. sandoru* sp. nov**.
5	Pronotal disc pale brown (Souma 2025: figs 3C, G, 4C, G, H, 5C, G, 6C, G, H); anterior margin of hemelytron strongly curved downward in apical half (Souma 2025: figs 7C, G, 8C, G, H, 9C, G, 10C, G, H); subcostal and discoidal areas of hemelytron united; Sc vein of hemelytron indistinct in apical part of dorsal view	**6**
–	Pronotal disc black (Souma 2025: figs 3D, F, 4D, F, 5D, F, 6D, F); anterior margin of hemelytron not or weakly curved downward in apical half (Souma 2025: figs 7D, F, 8D, F, 9D, F, 10D, F); subcostal and discoidal areas of hemelytron not united; Sc vein of hemelytron distinct in apical part of dorsal view	**8**
6	Paranotum without areolae in middle part, with areolae in remaining parts (Souma 2025: figs 3G, 4G, 5G, 6G, 14G); endemic to Chichijima Group	***O. majorcarinae* Guilbert, 2001**
–	Paranotum with areolae throughout its length (Souma 2025: figs 3C, 4C, H, 5C, 6C, H, 14C, H); endemic to Mukojima (Mukojima Group) or Hahajima (Hahajima Group) islands	**7**
7	Hood < 0.5× as wide as maximum width of head across compound eyes (Souma 2025: figs 3C, 4C, 5C, 6C, 14C); costal area wider than fused subcostal and discoidal areas (Souma 2025: figs 7C, 8C, 9C, 10C); endemic to Hahajima Island	***O. hisasuei* Souma, 2025**
–	Hood > 0.5× as wide as maximum width of head across compound eyes (Souma 2025: figs 4H, 6H, 14H); costal area narrower than fused subcostal and discoidal areas (Souma 2025: figs 8H, 10H); endemic to Mukojima Island	***O. mukojimensis* Souma, 2022**
8	Rostrum reaching middle part of mesosternum (Souma 2025: fig. 11D); paranotum with areolae throughout its length (Souma 2025: figs 3D, 4D, 5D, 6D, 14D); anterior margin of hemelytron not curved downward in apical half (Souma 2025: figs 7D, 8D, 9D, 10D); endemic to Hahajima Island	***O. inugusu* Souma, 2025**
–	Rostrum reaching posterior margin of mesosternum (Souma 2025: fig. 11F); paranotum without areolae in middle part, with areolae in remaining parts (Souma 2025: figs 3F, 4F, 5F, 6F, 14F); anterior margin of hemelytron weakly curved downward in apical half (Souma 2025: figs 7F, 8F, 9F, 10F); endemic to Chichijima Group	***O. kobugashi* Souma, 2025**

## Discussion

### Phylogenetic relationships within *Omoplax*

We found well-supported phylogenetic relationships within the genus *Omoplax*, excluding *O. mukojimensis* (Fig. 3). The following three pairs of *Omoplax* species that feed on the same or congeneric host plants are allopatrically distributed in island groups or islands and possess morphological characteristics not observed in the other two pairs (cf. Souma 2025): *O. desecta*, as identified based on previous studies, and *O. karubei* feeding on Rhaphiolepis indica var. tashiroi; *O. hisasuei* and *O. majorcarinae* feeding on Neolitsea sericeavar. aurata; and *O. inugusu* and *O. kobugashi* feeding on *Machilus* species. Furthermore, *O. mukojimensis*, which is allopatrically distributed on a different island group than *O. hisasuei* and *O. majorcarinae*, shares morphological characteristics with them (Souma 2025). However, the ML tree suggests that shared morphological characteristics and life histories not necessarily reflects a phylogenetic relationship within the genus *Omoplax*: (1) a sister relationship between *O. hisasuei* and *O. majorcarinae* was not supported; (2) a sister relationship between *O. inugusu* and *O. kobugashi* was not supported; (3) a sister relationship between *O. desecta* [= population in Chichijima Group of *O. desecta*, as identified based on previous studies] and *O. karubei* was supported; (4) a sister relationship between *O. hisasuei* and *O. sandoru* sp. nov. [= population in Hahajima Group of *O. desecta*, as identified based on previous studies] was well supported. In the genus *Stephanitis*, which resembles the genus *Omoplax* in morphological characteristics and feeding habits and is widely distributed throughout Japanese territories outside the Ogasawara Islands, multiple species that had been confused because of their morphological similarities (e.g., S. (Norba) aperta Horváth, 1912 and S. (N.) exigua) were not placed in the same monophyletic clade in the molecular phylogenetic analysis (Souma 2022b). Shared morphological characteristics and life histories do not seem useful for inferring the phylogenetic relationships within *Stephanitis* and its allied genera. To clarify the complete phylogenetic relationships within the genus *Omoplax*, additional individuals of *O. mukojimensis* must be collected.

### Speciation of endemic lace bugs

We aimed to clarify the species diversity and phylogenetic relationships within the genus *Omoplax* endemic to the Ogasawara Islands and did not conduct ancestral state reconstruction (ASR) and divergence time estimation in this study. Nevertheless, the results of this study shed light on the mechanism of speciation in endemic lace bugs. The ML tree indicated multiple host shifts to Rhaphiolepis indica var. tashiroi or Neolitsea sericeavar. aurata (Fig. 3). In addition, the sister relationship between *O. hisasuei* and *O. sandoru* sp. nov., which feed on different host plants and are distributed on the same island, can be hypothesized to be the result of speciation via a host shift. Conversely, *Calophyllum inophyllum*, which is widespread in the three island groups (Chichijima, Hahajima, and Mukojima) of the Ogasawara Islands, does not exhibit genetic structure among the islands because of past planting activities (Department of Forest Molecular Genetics, Forestry and Forest Products Research Institute 2015) and is considered an introduced species in these islands from tropical Asia, Polynesia, or Madagascar (Ohashi 2016). The host race feeding on *C. inophyllum* of *O. desecta*, which was observed in Chichijima Group, appears to exemplify recent host race formation, similar to that of the apple maggot *Rhagoletis pomonella* (Walsh, 1867) (Diptera, Tephritidae) for which has occurred a host shift from hawthorns to apples during the past few hundred years (Feder et al. 1988). The monophyly of this host race was well supported by the ML tree. Thus, host shift from R. indica var. tashiroi to *C. inophyllum* in *O. desecta* likely occurred only once recently. Furthermore, the distribution ranges of *O. desecta* and *O. karubei* are generally consistent with the genetic structure between the three island groups of R. indica var. tashiroi (cf. Department of Forest Molecular Genetics and Biotechnology, Forestry and Forest Products Research Institute 2017). The discovery of *O. karubei* on Nakodojima Island of Mukojima Group in the section above (Fig. 10; Suppl. material 2) did not confirm the sympatric distribution with *O. desecta*. The population of *O. desecta* on Nakodojima Island, for which host plant information is lacking, may consist only of the host race feeding on *C. inophyllum*, which was recently invaded. Consequently, the sister relationship between *O. desecta* and *O. karubei*, which typically feed on the same host plants and are distributed on different islands, can be hypothesized to be the result of speciation via geographic isolation. Future studies using ASR on other datasets, including all *Omoplax* species, are required to infer the mechanism of speciation within the genus *Omoplax*. The present study did not examine the genetic exchange and differentiation in detail between the two host races of *O. desecta* (Figs 1, 2). Further population genetic analyses of *O. desecta*, involving more localities and individuals, are necessary to understand the early stages of speciation in endemic insects on oceanic islands.

The divergence times of endemic terrestrial animal species on the Ogasawara Islands and species from other regions such as the Japanese Archipelago are estimated to have occurred during the Pliocene or early Pleistocene, when these islands remained above sea level continuously (Kaizuka 1977; Imaizumi and Tamura 1984; Miura et al. 2008; Sota et al. 2011; Huang et al. 2019; Nagata et al. 2021; Chen et al. 2026). Like other endemic terrestrial animals, endemic lace bugs may have diverged from those in other regions such as the Japanese Archipelago during the same geological era. However, in lace bugs endemic to the Ogasawara Islands, there are no specimens of *Acanthomoplax tomokunii* and *O. mukojimensis* from which sufficient DNA can be extracted. The genus *Acanthomoplax*, which comprises only *A. tomokunii*, is endemic to the Ogasawara Islands, similar to the genus *Omoplax* (Souma 2025). Future studies using phylogenetic analyses and divergence time estimation focusing on East Asian lace bugs, including all or most species of *Acanthomoplax*, *Omoplax*, and *Stephanitis*, which are allied with each other (Horváth 1912; Takeya 1962; Souma and Kamitani 2021), are required to determine the origin of lace bugs endemic to the Ogasawara Islands. Elucidating the mechanism of speciation and origin of lace bugs endemic to the Ogasawara Islands will deepen our understanding of the rapid adaptive radiation of insects on oceanic islands and enhance the value of these islands as a World Natural Heritage site.

## Conclusions

In this study, population genetic and phylogenetic analyses of the genus *Omoplax*, which is endemic to the Japanese oceanic islands (the Ogasawara Islands), based on genome-wide SNP data led to the description of an eighth species within the genus. Additionally, geographic isolation and host shift were hypothesized to be the mechanisms of speciation within *Omoplax*. However, how many times each of these mechanisms contributed to speciation within *Omoplax* remains unknown. Additionally, the origin of the endemic lace bugs on the Ogasawara Islands, including *Acanthomoplax*, which is morphologically similar to *Omoplax* (Souma and Kamitani 2021), remains unclear. Further elucidating the evolutionary history of these endemic lace bugs requires collecting samples of *A. tomokunii* and *O. mukojimensis* from which DNA can be extracted. For *O. mukojimensis*, elucidating its host plants is also necessary. Constructing a phylogenetic tree that comprehensively includes species of endemic lace bugs and their allies, *Stephanitis* (Horváth 1912; Takeya 1962; Souma and Kamitani 2021), together with ASR and divergence time estimation, will thoroughly clarify the adaptive radiation of lace bugs in the Ogasawara Islands.

## Supplementary Material

XML Treatment for Omoplax

XML Treatment for Omoplax desecta

XML Treatment for Omoplax karubei

XML Treatment for Omoplax sandoru

## Additional information

### Artificial Intelligence (AI) use

The authors accept full responsibility for the content of the manuscript, including the disclosure of any use of AI.

While preparing this paper, the authors used DeepL (https://www.deepl.com/ja/home) to assist with language, style, and writing. The authors reviewed and verified all content generated with AI assistance. The authors accept full responsibility for the content of the manuscript, including the disclosure of any AI usage.

### Author ORCIDs

J. Souma https://orcid.org/0000-0002-2238-5015

N. Nakahama https://orcid.org/0000-0003-3106-8289

### Data availability

The datasets supporting the conclusions of this study, excluding sequence reads, are included in the article and its supplementary information files. Sequence reads are available from the Sequence Read Archive of the DNA Data Bank of Japan (DDBJ) under the accession numbers PRJNA1477673 (BioProject) and SAMN60765438–SAMN60765489 (BioSample).

## Supplementary materials

Supplementary material 1 Sample IDs and DDBJ accession numbers for 52 specimens of eight lace bug species used for DNA extraction Data type xlsx https://binary.pensoft.net/file/1804594This dataset is made available under the Open Database License (http://opendatacommons.org/licenses/odbl/1.0/). The Open Database License (ODbL) is a license agreement intended to allow users to freely share, modify, and use this Dataset while maintaining this same freedom for others, provided that the original source and author(s) are credited.Author: Jun Souma

Supplementary material 2 Distribution and host plants of lace bugs occurring in the Ogasawara Islands, Japan Data type xlsx https://binary.pensoft.net/file/1804595This dataset is made available under the Open Database License (http://opendatacommons.org/licenses/odbl/1.0/). The Open Database License (ODbL) is a license agreement intended to allow users to freely share, modify, and use this Dataset while maintaining this same freedom for others, provided that the original source and author(s) are credited.Author: Jun Souma
